# Clinical diagnosis of Lewy body dementia

**DOI:** 10.1192/bjo.2020.44

**Published:** 2020-06-16

**Authors:** Ajenthan Surendranathan, Joseph P. M. Kane, Allison Bentley, Sally A. H. Barker, John-Paul Taylor, Alan J. Thomas, Louise M. Allan, Richard J. McNally, Peter W. James, Ian G. McKeith, David J. Burn, John T. O'Brien

**Affiliations:** Ajenthan Surendranathan, Department of Psychiatry, University of Cambridge, UK; School of Medicine, Dentistry and Biomedical Sciences, Queen's University Belfast, UK; Department of Psychiatry, University of Cambridge, UK; Translational and Clinical Research Institute, Newcastle University, UK; Institute of Neuroscience, Newcastle University, UK; Translational and Clinical Research Institute, Newcastle University, UK; Geriatric Medicine, University of Exeter, UK; Institute of Health and Society, Newcastle University, UK; Institute of Health and Society, Newcastle University, UK; Translational and Clinical Research Institute, Newcastle University, UK; Faculty of Medical Sciences, Newcastle University, UK; Department of Psychiatry, University of Cambridge, UK

**Keywords:** Dementia, clinical neurology, epidemiology, outpatient treatment, comorbidity

## Abstract

**Background:**

Lewy body dementia, consisting of both dementia with Lewy bodies (DLB) and Parkinson's disease dementia (PDD), is considerably under-recognised clinically compared with its frequency in autopsy series.

**Aims:**

This study investigated the clinical diagnostic pathways of patients with Lewy body dementia to assess if difficulties in diagnosis may be contributing to these differences.

**Method:**

We reviewed the medical notes of 74 people with DLB and 72 with non-DLB dementia matched for age, gender and cognitive performance, together with 38 people with PDD and 35 with Parkinson's disease, matched for age and gender, from two geographically distinct UK regions.

**Results:**

The cases of individuals with DLB took longer to reach a final diagnosis (1.2 *v*. 0.6 years, *P* = 0.017), underwent more scans (1.7 *v*. 1.2, *P* = 0.002) and had more alternative prior diagnoses (0.8 *v*. 0.4, *P* = 0.002), than the cases of those with non-DLB dementia. Individuals diagnosed in one region of the UK had significantly more core features (2.1 *v*. 1.5, *P* = 0.007) than those in the other region, and were less likely to have dopamine transporter imaging (*P* < 0.001). For patients with PDD, more than 1.4 years prior to receiving a dementia diagnosis: 46% (12 of 26) had documented impaired activities of daily living because of cognitive impairment, 57% (16 of 28) had cognitive impairment in multiple domains, with 38% (6 of 16) having both, and 39% (9 of 23) already receiving anti-dementia drugs.

**Conclusions:**

Our results show the pathway to diagnosis of DLB is longer and more complex than for non-DLB dementia. There were also marked differences between regions in the thresholds clinicians adopt for diagnosing DLB and also in the use of dopamine transporter imaging. For PDD, a diagnosis of dementia was delayed well beyond symptom onset and even treatment.

Lewy body dementia, consisting of dementia with Lewy bodies (DLB) and Parkinson's disease dementia (PDD), is the second most common neurodegenerative dementia in older people, and comprises 15–20% of cases of dementia in pathological studies.^1,2^ However, clinically the prevalence is much lower, with DLB prevalence reported to be 4.2–5%^3,4^ of all patients with dementia and PDD prevalence is reported to be 3.6%.^5^ In this study we analysed the diagnostic pathways of patients with DLB and PDD, to understand if difficulties in diagnosis contribute to the divergence in LBD prevalence between clinical and pathological samples.

## Method

An initial survey of the prevalence in secondary care clinical services of patients diagnosed with DLB and those with PDD was carried out in two geographical areas of UK – East Anglia and the North East of England (the ‘North East’) – and has been previously reported.^6^ The regions and underlying services were chosen by the research team in order to compile a cohort generalisable to that seen in routine clinical practice and included those serving both urban populations and mixed urban and rural populations. For assessing DLB, the survey investigated the frequency of the diagnosis as a proportion of all patients with dementia in nine old-age psychiatry/memory clinic services across four National Health Service (NHS) hospital trusts. The majority (seven) of the services were secondary care organisations, whereas the remaining two consisted of a tertiary memory clinic combining psychiatry and neurology expertise in East Anglia, as well as a tertiary DLB clinic in the North East, reviewing individuals diagnosed with DLB from a secondary care centre.

For assessing the proportion of PDD diagnosis in people with Parkinson's disease, five Parkinson's disease or movement disorder clinics, each from a separate NHS trust (three in East Anglia, two in the North East) were sampled. These consisted of three combined geriatric medicine and neurology services and two geriatric medicine only services, serving two urban and three mixed urban and rural populations. None of these services incorporated specialist tertiary clinics.

For DLB, all new cases of patients referred to and assessed within selected services in an 18-month period during 2013 and 2014, were surveyed for diagnoses made. This entailed a brief review of the medical records of each patient from that service to detect if dementia was diagnosed. If so, further demographic and diagnostic details were recorded. Patients were considered to have a DLB diagnosis if their last diagnosis in the medical records in the screening period was either ‘probable’ or ‘possible’ DLB or mixed dementia with DLB specifically mentioned. The study was carried out prior to the publication of the 2017 consensus criteria for DLB,^7^ hence the medical notes reflected the use by clinicians of the earlier 2005 criteria^8^ and we have therefore referred to the 2005 criteria in the analysis of the results.

For PDD, all patients seen in the stated services in an 18-month period within 2014 and 2015, aged 65 and over, were surveyed for whether a Parkinson's disease diagnosis was made. Demographic and diagnostic details were then collected for such patients. Patients were recorded as having PDD where the records specifically stated ‘dementia’ as a diagnosis. The term ‘cognitive impairment’ or similar terms were not sufficient. The 2007 Movement Disorder Society (MDS) criteria^9,10^ were the relevant criteria deemed to have been applied by clinicians in the diagnosis of PDD.

### Medical notes review

Patients identified in the initial prevalence survey as diagnosed with DLB or, in the case of the PDD survey, PDD, were approached for written consent to carry out a detailed analysis of their diagnostic pathway, except if (a) it was deemed the approach was unsuitable on clinical grounds, (b) they could not be contacted, or (c) they had died.

Once consent was obtained, a matched comparison/control patient was identified for that participant from the next consecutive patient with non-DLB dementia or non-dementia Parkinson's disease (as appropriate) seen in the same service who satisfied the matching criteria (below) and they were approached for written consent. Patients with DLB were matched according to gender, age at dementia diagnosis (+/– 5 years) and Mini-Mental State Examination (MMSE)^11^ score at time of diagnosis (with severe: 0–9; moderate: 10–20; or mild: ≥21).

Patients with PDD were matched according to age at referral for Parkinson's disease symptoms (+/– 5 years) and gender. If the next such potential comparison patient declined or was not suitable, the following patient that matched criteria was identified, and so on until a comparison patient was recruited.

For patients with DLB, a PDD diagnosis (in accordance with the 2007 MDS criteria) was an exclusion criterion in the recruitment of control participants. For the Parkinson's disease (non-dementia) comparison patients, treatment with anti-dementia drugs during the 18-month survey period was an exclusion criterion, since prescription of these drugs would suggest significant cognitive impairment and possible *de facto* dementia despite dementia not being formally diagnosed by the clinician in the medical records, hence such participants would not be suitable control patients.

### Recruitment

With respect to the patients with DLB, 207 were identified in the prevalence study. Of these, 102 were not approached: 54 had died before we could approach, 6 were deemed unsuitable for approach by the research team (for example they had already stated that they were not interested in participating in research), 24 were deemed unsuitable for approach by the clinical team and 18 were not contactable. Of the 105 that were approached, 30 declined. One further patient was excluded following review of the diagnosis by the expert panel, leaving 74 participants in the analysis (the DLB group).

For the patients with PDD, 161 were identified in the prevalence study. From this group, 108 were not approached, this included: 12 who had died, 3 deemed unsuitable for approach by the research team, 15 deemed unsuitable for approach by the clinical team, 4 who were not contactable and 74 who were not considered for approach. Of the 53 remaining, 15 declined participation, leaving 38 recruited patients (the PDD group).

One DLB participant could not be matched despite an extensive search, leaving 73 matched comparison participants (the non-DLB group). Of the 38 patients with PDD recruited, 3 were similarly unmatched, leaving 35 comparison patients (the Parkinson's disease group).

Once written informed consent was obtained, a case report form was completed for each participant based on the patient's medical records, collating the details of their diagnostic and management pathway including clinical features, investigations, clinical appointments and medications prescribed from the time of their initial referral for their symptoms to the end of the 18-month screening period. A panel of expert clinicians subsequently reviewed each case report form to verify the diagnosis. The 2005 consensus criteria^8^ for DLB and the 2007 MDS criteria^9,10^ for PDD were applied as appropriate to each participant, including the control participants with the aim of excluding participants from the analysis if the LBD diagnosis were incorrect in the participants in the two LBD groups (i.e. DLB and PDD groups) or if participants with LBD were identified in the two control groups (i.e. non-DLB and Parkinson's disease groups).^8–10^

One matched comparison patient for the DLB group, diagnosed by the clinical team with Alzheimer's disease was subsequently excluded as they were deemed by the expert panel to have PDD, hence leaving 72 control patients in that group. The experts achieved consensus and agreed with all other diagnoses.

### Statistical analysis

Data were analysed using the Statistical Package for Social Sciences software version 25 (SPSS; IBM Corporation, USA). Differences in demographic and clinical data were assessed using either *t*-tests, analysis of variance (ANOVA), or rank-sum tests (Mann–Whitney *U*) as appropriate for continuous variables and χ^2^-test for categorical data. Pearson's correlation was used to assess linear associations for continuous variables. For each test statistic, a probability value (*P*) of <0.05 was regarded as significant.

### Ethical approval

The authors assert that all procedures contributing to this work comply with the ethical standards of the relevant national and institutional committees on human experimentation and with the Helsinki Declaration of 1975, as revised in 2008. All procedures involving human patients were approved by regional NHS Research Ethics Committee (NRES Committee North East—Newcastle & North Tyneside 1; Reference 13/NE/0268).

## Results

### Demographics

Patients and controls were well matched for age at time of referral and gender (see Table 1), as would be expected from the matching process used. The non-DLB group consisted of 48 participants with Alzheimer's disease, 7 with vascular and 17 with mixed (Alzheimer's disease and vascular) dementia.

**Table 1:** Demographics: comparison of gender, age at referral and disease duration for participants

	DLB group	Non-DLB group	Statistics, DLB versus non-DLB groups			PDD group	Parkinson's disease group	Statistics, PDD group versus Parkinson's disease group		
			*t*-test	χ^2^-test	*P*			*t*-test	χ^2^-test	*P*
Participants, *n*	74	72	–	N/A	N/A	38	35	–	N/A	N/A
Age, years: mean (s.d.)	77.6 (8.4)	77.2 (8.0)	0.31		0.76	70.7 (7.0)	70.4 (7.2)	0.19	–	0.85
Gender, male/female: *n*	44/30	42/30		0.19	0.89	28/10	26/9	–	0.003	0.95
MMSE score, mean (s.d.)	21.2 (5.3)	20.3 (4.9)	1.04		0.30					
Disease duration, from onset of cognitive symptoms to final diagnosis (years): mean (s.d.).	2.9 (2.8)	2.3 (2.6)	1.26	–	0.21	–	–	–	–	–
Duration of Parkinson's disease, from diagnosis to end of screening period (years): mean (s.d.).	–	–	–	–	–	9.4 (5.7)	6.6 (3.9)	−2.3	–	0.024

MMSE, Mini-Mental State Examination.

### Comparison of diagnostic pathways in DLB

A comparison of the diagnostic pathways of the DLB and non-DLB dementia groups was carried out (see Table 2). Before a final diagnosis was made, those in the DLB group received significantly more alternate diagnoses, clinical assessments at home and imaging tests (including dopaminergic ^123^I-N-ω-fluoropropyl-2beta-carbomethoxy-3beta-4-iodophenyl nortropane (^123^I-FP-CIT) brain single-photon emission computerised tomography (SPECT) (FP-CIT) imaging) than those in the non-DLB group.

**Table 2:** Comparison of the diagnostic pathways for dementia with Lewy bodies (DLB) group versus non-DLB group^a^

	DLB group, mean (s.d.) range	Non-DLB group, mean (s.d.) range	Student's *t*-test	*P*
Number of diagnoses before final diagnosis	0.8 (0.9) 0–4	0.4 (0.7) 0–3	3.08	0.002
Time between first secondary care appointment and final diagnosis (years)	1.2 (1.8) 0–8	0.6 (1.0) 0–5	2.42	0.017
Number of imaging tests before final diagnosis (including FP-CIT scan)	1.7 (1.0) 0–5	1.2 (0.8) 0–4	3.09	0.002
Number of clinical assessments at home before final diagnosis	3.9 (7.3) 0–40	1.8 (2.5) 0–15	2.31	0.02
Number of clinic appointments before final diagnosis	2.6 (5.7) 0–30	1.5 (2.2) 0–13	1.45	0.15

FP-CIT, ^123^I-N-ω-fluoropropyl-2beta-carbomethoxy-3beta-4-iodophenyl nortropane brain single-photon emission computerised tomography.

a. How the DLB and non-DLB group reached their final diagnosis was compared, including the number of alternative diagnoses received before their final diagnosis.

When ‘mild cognitive impairment’ (MCI), which accounted for about a fifth of these alternate initial diagnoses across both groups, was removed the differences were even greater with a mean of 0.65 alternate prior diagnoses for the DLB group compared with 0.17 for the non-DLB group (*t* = 4.27, *P* < 0.001).

In addition, the proportion of the DLB group receiving an alternate diagnosis prior to their final diagnosis was higher than in the non-DLB group (51% (38/74) *v*. 31% (22/72), χ^2^ = 6.5, *P* = 0.01). Removing MCI diagnoses from both groups again led to even greater differences, with 46% (34/74) of the DLB group but only 13% (9/72) of the non-DLB group receiving an alternate prior diagnosis (χ^2^ = 19.6, *P* < 0.001). The DLB group also had a significantly longer time period, on average 1.2 years compared with 0.6 years, between their first appointment in secondary care and the date of their final diagnosis.

The mean time between the date of final diagnosis and initiation of treatment for dementia (such as donepezil) was also significantly different between groups. Treatment on average was started before a final diagnosis in the DLB group and afterwards in the non-DLB group (a mean of 0.30 years before in the DLB group and 0.15 years after in the non-DLB group; *t* = −3.11, *P* = 0.002). We did not, however, find a significant difference between the time of onset of cognitive impairment and initiation of treatment (mean of 2.4 years for both the DLB and non-DLB groups; *t* = 0.16, *P* = 0.87).

**Fig. 1:**
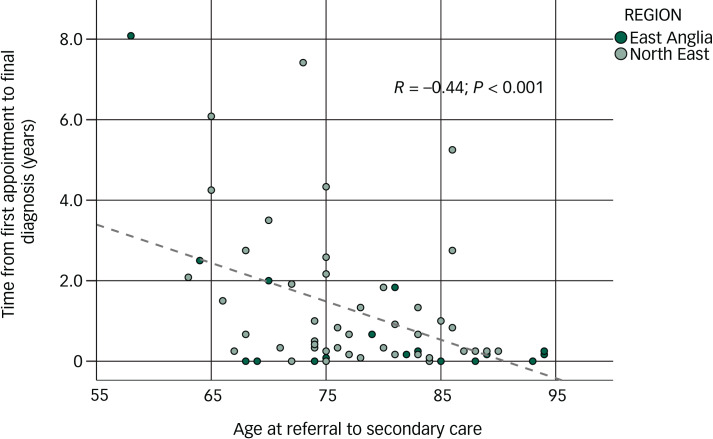
Correlation between age of participants with dementia with Lewy bodies (DLB) and time from first appointment to final diagnosis.

We also found that the younger the patient, the longer it took from first appointment to final diagnosis, in the DLB group (Pearson's, *R* = −0.44, *P* < 0.001; see Fig. 1) and this was more so than in the non-DLB group (Pearson's, *R* = −0.21, *P* = 0.08).

Relationship between age, and time from first appointment to final diagnosis, in patients with DLB.

### Comparison of symptoms in DLB

When the two geographical regions were compared (see Table 3), at the time of diagnosis, a significantly higher number of core clinical features,^8^ but a significantly lower number of suggestive features were recorded in the participants with DLB in East Anglia compared with those in the North East. There were also a significantly higher number of FP-CIT scans (31 compared to 1) carried out in the North East, and a high proportion of these were abnormal (77%).

**Table 3:** Regional differences in dementia with Lewy bodies (DLB) diagnosis^a^

	North East	East Anglia	*t*-test	χ^2^-test	*P*
Core features of DLB at time of diagnosis, mean (s.d.) range	1.5 (0.9) 0–3	2.1 (0.9) 0–3	–2.78	–	0.007
Suggestive features of DLB at time of diagnosis, including FP-CIT scan, mean (s.d.) range	0.8 (0.8) 0–2	0.4 (0.5) 0–1	2.63	–	0.011
Abnormal FP-CIT scan prior to diagnosis, *n*	24	1	–	12.9	0.001
FP-CIT scan prior to diagnosis (including normal), *n*	31	1	–	20.6	<0.001
Total diagnostic features (core and suggestive) of DLB at time of diagnosis, mean (s.d.)	2.4 (1.0)	2.6 (0.9)	−0.80	–	0.42
Time between first secondary care appointment and final diagnosis in years, mean (s.d.) range	1.4 (1.7) 0–7	0.9 (1.9) 0–8	1.03	–	0.31

FP-CIT, ^123^I-N-ω-fluoropropyl-2beta-carbomethoxy-3beta-4-iodophenyl nortropane brain single-photon emission computerised tomography.

a. Core and suggestive features as set out in the 2005 diagnostic criteria.^8^

### Diagnostic threshold in DLB

**Fig. 2:**
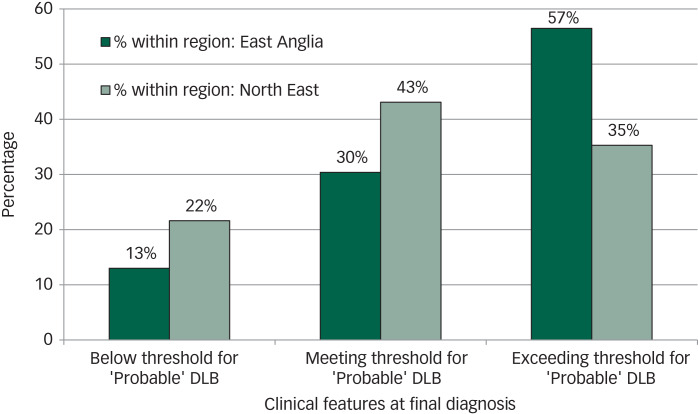
Diagnostic threshold in dementia with Lewy bodies (DLB).

The majority of participants with DLB (57%, 13/23) in East Anglia exceeded the threshold for ‘probable’ DLB as set out in the 2005 criteria^8^ (namely more than two core features or one core feature and a suggestive feature) at the time of final diagnosis, whereas only 35% (18/51) exceeded this threshold in the North East (see Fig. 2).

Regional differences in diagnostic threshold for DLB diagnosis with respect to clinical features at the time of final diagnosis.

### Comparison of imaging and comorbidities in DLB

Prior to diagnosis, the DLB group had a similar number of computed tomography (CT) head scans and SPECT perfusion brain imaging to those in the non-DLB group, but more magnetic resonance imaging (MRI) scans and FP-CIT scans (see Table 4).

**Table 4:** Comparison of imaging between groups^a^

	DLB group, mean (s.d.)	Non-DLB group, mean (s.d.)	*t*-test	*P*
CT, head	0.73 (0.7)	0.81 (0.6)	−0.71	0.77
MRI, head	0.32 (0.5)	0.17 (0.4)	2.1	0.04
FP-CIT	0.45 (0.5)	0.03 (0.2)	6.5	<0.001
Perfusion brain SPECT	0.14 (0.3)	0.15 (0.4)	−0.29	0.78

DLB, dementia with Lewy bodies; CT, computed tomography; MRI, magnetic resonance imaging; FP-CIT, ^123^I-N-ω-fluoropropyl-2beta-carbomethoxy-3beta-4-iodophenyl nortropane brain single-photon emission computerised tomography; SPECT, single-photon emission computerised tomography.

a. Comparison of the mean number of each brain imaging test per patient, carried out prior to the final diagnosis in each group.

The vast majority of patients with dementia of both types were diagnosed by old age psychiatrists (84% of the DLB group (62/74) and 93% of the non-DLB group (67/72), with a slightly higher proportion of those in the DLB group than in the non-DLB group diagnosed by neurologists (11% *v*. 3%, 8/74 *v.* 2/72). The remaining patients were diagnosed by geriatricians.

The DLB group also experienced significantly higher rates of repeated falls, urinary incontinence, non-visual hallucinations and delusions compared with the non-DLB dementia group (see Table 5).

**Table 5:** Comparison of non-core features of dementia with Lewy bodies (DLB) in the DLB and non-DLB groups

	DLB group, % (*n*)	Non-DLB group, % (*n*)	χ^2^-test	*P*
Repeated falls	63.9 (46/72)	34.5 (19/55)	10.7	0.001
Constipation	54.5 (30/55)	39.5 (17/43)	2.2	0.14
Urinary incontinence	64.4 (38/59)	36.8 (14/38)	7.1	0.008
Orthostatic hypotension	44.7 (21/47)	24.1 (7/29)	3.3	0.07
Depression	40.0 (28/70)	39.7 (27/68)	<0.1	0.97
Hallucinations (non-visual)	31.3 (21/67)	3.3 (2/61)	17.1	<0.001
Delusions	37.7 (26/69)	6.3 (4/63)	18.4	<0.001

### Dementia symptoms before diagnosis in PDD

Analysis of the case notes for the PDD group revealed that 46% (12/26 participants with PDD, as for a further 12 participants with PDD data were missing) had impaired activities of daily living (ADLs) because of cognitive impairment prior to their diagnosis of dementia. The mean intervening time from ADL impairment being recorded and dementia diagnosis was 1.5 years. Furthermore, 57% (16/28 of the PDD group) had cognitive impairment in multiple domains with a mean onset time period of 2 years prior to dementia diagnosis. In addition, 38% (6/16 of the PDD group) had both multidomain cognitive impairment and as a consequence, impaired ADLs, prior to a diagnosis of dementia, with a mean time of 1.4 years before such dementia diagnosis.

### Treatment before diagnosis in PDD

In total, 39% (9/23) of the PDD group were started on anti-dementia drugs in the form of rivastigmine or donepezil before a diagnosis of dementia, with the mean time of starting the treatment before diagnosis being 1 year and 9 months.

### Participants with symptomatic Parkinson's disease not diagnosed with dementia

Patients without a diagnosis of PDD but nevertheless being treated with anti-dementia medications (such as rivastigmine) within the screening period had been excluded from being in the Parkinson's disease control group. Nevertheless, five of the Parkinson's disease control group were found to have received anti-dementia medications (both initiated and withdrawn) before the onset of the screening period. One such patient who had received rivastigmine was noted to have had visual hallucinations, which may have been an indication for starting that drug as it can be initiated for treatment of psychosis. None of the other patients on anti-dementia treatment were recorded to have any other features of psychosis.

In addition, two of the Parkinson's disease group had cognitive impairment that affected ADLs and six of patients in this group had cognitive impairment in multiple domains.

### Clinicians making the diagnosis

Where data was available (for 34 in the PDD group and 32 in the Parkinson's disease group), old age psychiatrists were found to have made the dementia diagnoses in 16 patients in the PDD (47%) group, neurologists in 3 (9%), geriatricians in 13 (38%), a Parkinson's disease nurse specialist in 1 (3%) and a general practitioner in 1 (3%). For the Parkinson's disease group, the diagnosis of Parkinson's disease was made by neurologists in 9 (28%) and geriatricians in 23 (72%) patients.

### Symptoms and comorbidities in the PDD group compared with the Parkinson's disease group

The PDD group had more visual hallucinations (86% *v*. 28% (31/36 *v.* 8/29), χ^2^ = 22.9, *P* < 0.001) and cognitive fluctuations (75% *v*. 11% (21/28 *v.* 3/27), χ^2^ = 22.8, *P* < 0.001) than the Parkinson's disease group. Although rapid eye movement sleep behaviour disorder was also more common, there was no statistically significant difference (53% *v*. 33% (18/34 *v.* 10/30), χ^2^ = 2.5, *P* = 0.12).

## Discussion

### DLB diagnostic pathway

This study reveals delays in the diagnosis of DLB compared with non-DLB dementia. Patients with DLB experienced a longer length of time between their first appointment and receiving a final diagnosis and had more alternative diagnoses ascribed within this time. The DLB group also had a higher mean number of home visits before a final diagnosis and a greater number of imaging tests. We also found that the lower the age of the participants with DLB, the longer the time to final diagnosis.

In addition, our results also show that the time to initiation of anti-dementia drugs after the onset of cognitive symptoms was not significantly different between the groups. Nevertheless, the mean time between the date of final diagnosis and initiation of treatment was significantly different, again suggesting difficulty with identifying dementia subtype rather than dementia itself.

The pathway to diagnosis is hence longer and more challenging for patients with DLB. The results of this study are consistent with a retrospective study of caregiver experience of patients with LBD,^12^ which found two-thirds of patients saw more than three doctors before an LBD diagnosis was made and a third needed more than six clinic visits. In addition, in 78% of people a diagnosis of another disorder was made first. However, that study was entirely based on caregiver reports with no independent or objective information to verify the diagnostic pathway, and also without any comparisons with patients with non-LBD. As this was a retrospective study, it could also have been affected by recall bias, especially where the experience was mostly negative.

The importance of correctly diagnosing DLB has been highlighted in a review^13^ that identified far-reaching consequences of having LBD that may not be appreciated without a diagnosis. The gravest danger being the inadvertent use of anti-psychotics, which can be fatal in DLB if neuroleptic malignant syndrome is triggered but can more commonly lead to worsening of their debilitating movement disorder. The latter may not even be realised by doctors or patients if it is thought of as progression of dementia. Patients may also not receive symptomatic treatment for bradykinesia or rigidity. Hence a delay in achieving the correct diagnosis could be severely detrimental to the patient's care.

Patients with LBD also report lower quality of life than patients with Alzheimer's disease, often because of autonomic and neuropsychiatric symptoms.^14^ Higher caregiver stress was also reported compared with patients with Alzheimer's disease, in association with neuropsychiatric symptoms, as well as impaired ADLs.^15,16^ High levels of caregiver stress have also been identified in LBD as a whole and this was associated with behavioural problems and impaired ADLs, together with isolation,^17^ although that study did not compare LBD with other dementia subtypes. There is also evidence for a higher mortality risk in DLB than Alzheimer's disease.^13^

The timely diagnosis of DLB is therefore important for providing the necessary clinical care as well as support to the patient and their caregiver.

### Regional variation in DLB diagnosis

We found regional variations in the UK in how DLB was diagnosed, in particular the thresholds clinicians required for a DLB diagnosis and also in their use of FP-CIT scans. Clinicians in East Anglia appeared to require higher levels of evidence to make a diagnosis of DLB, which may explain the previously reported lower rates of diagnosis of DLB in this region compared with the North East (3.3% *v*. 5.6%).^6^ Such differences in the diagnostic approach of clinicians highlights the need for a more standardised approach.

The frequent use of FP-CIT scans in the North East compared with an almost complete absence of their use in East Anglia (31 *v*. 1 scans used, respectively), may also partly explain these differences in diagnostic rates. FP-CIT imaging has been shown using autopsy validation to have high rates of sensitivity (>80%) and specificity (>90%) for DLB.^18,19^ An abnormal FP-CIT scan is a suggestive feature in the 2005 criteria^8^ and an indicative biomarker in the updated 2017 criteria.^7^ The reasons why clinicians in East Anglia are not utilising this form of imaging is unclear. The benefits of FP-CIT scans in patients with ‘possible’ DLB has previously been reported^20^ – an abnormal scan can increase the likelihood of clinicians diagnosing ‘probable’ DLB, as only one additional criterion is required: a single core feature. Clinicians may have also been more confident making a diagnosis with an abnormal scan result rather than purely on clinical grounds. Indeed, seven patients with DLB were diagnosed based on an abnormal FP-CIT scan and a single core feature in the North East, whereas there were no individuals in East Anglia diagnosed based on an abnormal FP-CIT scan and a single core feature. Hence the infrequent use of FP-CIT scans may be an important factor in the lower rates of diagnosis of DLB in East Anglia.

The higher threshold set by clinicians in East Anglia is despite the excellent specificity of the 2005 diagnostic criteria. A recent meta-analysis evaluated the 2005 DLB diagnostic criteria for identifying a ‘probable’ DLB diagnosis and reported a sensitivity of 88.3% and specificity of 80.8%, although there were only three underlying studies that all focused on later stages (>3 years after onset) of the disease.^21^ Nevertheless the high specificity recorded suggests setting a higher threshold than the 2005 consensus criteria is unlikely to increase the diagnostic accuracy, but would reduce the numbers detected, by diminishing the sensitivity.

Together, the delays in achieving the correct diagnosis and the variations in clinical thresholds set by clinicians for the diagnosis of DLB could be contributing to the differences in the prevalence of DLB reported in clinical and pathological studies. These results also hint at potentially missed cases of people with DLB, if for example further investigations are not pursued to eventually rectify an erroneous diagnosis or if clinicians set too high a threshold for diagnosing DLB.

### PDD diagnostic pathway

This study also shows that a diagnosis of dementia is often delayed in patients with Parkinson's disease. The diagnostic criteria for PDD^9^ requires impairment in two cognitive domains plus impairments in ADLs because of cognitive impairment, in the context of an insidious dementia syndrome. In this study we found that symptoms satisfying dementia in Parkinson's disease were identified in the medical records by clinicians, yet a diagnosis of dementia was not made until much later. We also found that many patients were being treated for dementia before receiving the diagnosis, again suggesting clinicians were aware such patients had symptoms consistent with dementia but delayed making a formal diagnosis. The results of this study also suggest that some patients with Parkinson's disease in the control group had features of dementia in Parkinson's disease recorded in their clinical notes and may have even been treated for dementia but were not being diagnosed as such.

Such a delay in the diagnosis of dementia in Parkinson's disease may again lead to a lower rate of diagnosis clinically. The results of our earlier prevalence study in the same regions found only 9.7% of patients with Parkinson's disease had been diagnosed with dementia^6^ – much lower than a meta-analysis that showed prevalence of dementia in Parkinson's disease to be 24.5%.^5^ A longitudinal study by Hely and colleagues that observed patients with Parkinson's disease over 20 years from diagnosis, found 83% of survivors developed dementia and 75% of those who had not survived to 20 years had also developed dementia.^22^ The study noted neurologists were more likely to underestimate than overestimate the prevalence of dementia in patients with Parkinson's disease, and recommended that dementia be actively sought and excluded rather than assumed to be absent.

A delay in the diagnosis of dementia has important implications for patients and their caregivers. A diagnosis allows for the provision of support services that they would not otherwise be able to access. Dementia also leads to loss of insight, poor judgement and difficulties in financial decision-making, together with impaired driving skills, among other difficulties,^23^ which can be considered and addressed once a diagnosis is formerly made.

Hely and colleagues suggest brief regular assessments throughout the disease course to detect cognitive decline.^22^ Instruments such as an assessment toolkit may aid clinicians in this regard. For patients with Parkinson's disease, who typically have regular follow-ups to assess their movement disorder, this would not mean an increase in clinic appointments.

### PDD symptoms

Visual hallucinations and fluctuating cognition were noted to be present significantly more in the PDD group (86% and 75%, respectively) than the Parkinson's disease control group (28% and 11%), suggesting these clinical features may have been used by clinicians as surrogate markers to make a dementia diagnosis – they are not part of the MDS criteria.^9^ Both are core features of DLB, which shares many of the pathological features of PDD,^24^ hence it is possible clinicians are making the dementia diagnoses with this in mind. Visual hallucinations are less frequent in PDD than DLB, but have been found to be a strong predictor for the onset of dementia.^25^ In addition, fluctuations in cognition have been reported at a similar frequency in patients with DLB and those with PDD but were not found at all in patients with Parkinson's disease.^26^

### Clinicians making the diagnosis

This study also found that LBD was diagnosed primarily by psychiatrists, irrespective of whether the initial referral to specialist services was to neurology or psychiatry. Although geriatricians and neurologists made the diagnoses of Parkinson's disease, a dementia diagnosis was made in the main by old age psychiatrists with geriatricians a close second. Patients with Parkinson's disease are regularly followed up by neurologists and geriatricians in the UK. Both sets of clinicians would be able to make a dementia diagnosis, but the study results suggest neurologists referred patients with Parkinson's disease to psychiatry services for a diagnosis of PDD. DLB diagnoses were also mainly made by psychiatrists; however, unlike patients with PDD, most patients with DLB were seen by psychiatrists initially.

### Limitations

The study is a retrospective study that could predispose to recall bias, however, the data was collected from contemporaneously written medical records rather than being based on recall by patients or clinicians. A further limitation is the lack of demographic data for those identified in the prevalence survey but not recruited for analysis, which may affect the generalisability of the results. Another potential limitation is that the diagnoses of the patients were not validated by autopsy, which is the gold standard for diagnosis. Nevertheless, all case report forms were verified by an expert panel assessing each patient's case with reference to the relevant diagnostic criteria.

### Implications

This study reveals delays in the diagnosis of both DLB and PDD. The pathway to diagnosis for patients with DLB is longer and more complex and some clinicians appear to set a high threshold for making a diagnosis. In PDD, there appears to be a lag in the diagnosis, beyond the onset of symptoms of dementia. Together these findings may explain why LBD is considerably under-recognised clinically compared with its frequency in autopsy studies of dementia and suggests many patients with LBD are not receiving an appropriate diagnosis and consequently neither the support nor the treatment that they desperately need.

## Data Availability

All authors had full access to the data.
